# Long-term occupational exposures on disability-free survival and mortality in older adults

**DOI:** 10.1093/occmed/kqad105

**Published:** 2023-11-08

**Authors:** S M Alif, G P Benke, H Kromhout, R Vermeulen, C Tran, K Ronaldson, K Walker-Bone, R Woods, L Beilin, A Tonkin, A J Owen, J J McNeil

**Affiliations:** School of Public Health and Preventive Medicine, Monash University, Melbourne, Victoria 3004, Australia; School of Health Sciences, The University of Melbourne, Melbourne, Victoria 3000, Australia; Institute of Health and Wellbeing, Federation University Australia, Berwick, Victoria 3806, Australia; School of Public Health and Preventive Medicine, Monash University, Melbourne, Victoria 3004, Australia; Environmental Epidemiology Division, Institute for Risk Assessment Sciences, Utrecht University, 3584 CS Utrecht, The Netherlands; Environmental Epidemiology Division, Institute for Risk Assessment Sciences, Utrecht University, 3584 CS Utrecht, The Netherlands; School of Public Health and Preventive Medicine, Monash University, Melbourne, Victoria 3004, Australia; School of Public Health and Preventive Medicine, Monash University, Melbourne, Victoria 3004, Australia; School of Public Health and Preventive Medicine, Monash University, Melbourne, Victoria 3004, Australia; School of Public Health and Preventive Medicine, Monash University, Melbourne, Victoria 3004, Australia; School of Medicine, The University of Western Australia, Perth, Western Australia 6009, Australia; School of Public Health and Preventive Medicine, Monash University, Melbourne, Victoria 3004, Australia; School of Public Health and Preventive Medicine, Monash University, Melbourne, Victoria 3004, Australia; School of Public Health and Preventive Medicine, Monash University, Melbourne, Victoria 3004, Australia

## Abstract

**Background:**

The impact of long-term occupational exposures on health in older adults is increasingly relevant as populations age. To date, no studies have reported their impact on survival free of disability in older adults.

**Aims:**

We aimed to investigate the association between long-term occupational exposure and disability-free survival (DFS), all-cause mortality and cause-specific mortality in initially healthy older adults.

**Methods:**

We analysed data from 12 215 healthy participants in the ASPirin in Reducing Events in the Elderly (ASPREE) study whose mean age was 75 years. Their work history was collated with the ‘ALOHA-plus JEM’ (Job Exposure Matrix) to assign occupational exposures. The primary endpoint, DFS, was a composite measure of death, dementia or persistent physical disability. The secondary endpoint, mortality, was classified according to the underlying cause. Cox proportional hazard models were used to calculate hazard ratios and 95% confidence intervals, adjusted for confounders.

**Results:**

A total of 1835 individuals reached the DFS endpoint during the median 4.7 years follow-up period. Both ever-high and cumulative exposure to all dusts and all pesticides during a person’s working years were associated with reduced DFS. Compared to no exposure, men with high exposure to dusts and pesticides had a reduced DFS. Neither of these exposures were significantly associated with all-cause mortality. Men with high occupational exposure to solvents and women exposed to dusts experienced higher all-cause and cancer-related mortality.

**Conclusions:**

Long-term occupational exposure to all dusts and pesticides was associated with a reduced DFS and increased mortality in community-dwelling healthy older adults.

## Teaser

Long-term occupational exposure may affect health in older age. Until now, no studies have investigated their influence on disability-free survival. This study analysed the ALSOP data from healthy older individuals, and identified that ever-high and cumulative exposure to dust and pesticides was associated with reduced disability-free survival after 70 years of age. However, no such association was observed with all solvent or metal exposure, suggesting that exposure to dust and pesticides may pose distinct risks.

**Key learning points**

**What is already known about this subject:**

- Occupational exposures during working life may affect health in older age.
- Little is known about occupational exposures and risk of disability-free survival in the elderly.

**What this study adds:**

- Ever-high and cumulative exposure to all dust and pesticides was associated with reduced disability-free survival.
- Men exposed to ever-high solvent exposure and women with ever-high all-dust exposure had increased all-cause mortality.

**What impact this may have on practice and policy:**

- Clinicians should recognize previous occupational exposure as a risk factor for impaired health in post-retirement years.
- The data suggest that all-dust and pesticide exposure may adversely affect healthy survival.

## Introduction

As life expectancy increases, a social and economic imperative is maintaining the health and independence of the ageing population [1]. Previous research has reported a range of ways in which a person’s occupation might affect later health [2]. A recent global burden of disease study found that occupational exposures, including dust, gases, fumes, noise and ergonomic factors, contribute to a 16% increase in all-cause mortality and disability-adjusted life years [3]. However, from a community perspective, the degree to which occupations held during working life influence health in the post-retirement period has not been well established.

A series of earlier population-based studies have demonstrated the effects of long-term occupational exposures on all-cause and cause-specific mortality. For example, epidemiological studies of workers in the textile [4], petroleum industries [5] and meat working [6] industries have reported increased all-cause mortality. Specific exposures implicated include solvents, metals and dust exposure [7]. In general, occupational exposure to carcinogenic substances is amongst the highest predictors of future mortality in high-income countries [3]. Pooled analysis from different studies also found an increased mortality risk due to occupational exposures [8,9]. However, the comparability of the existing literature is limited by the various exposure assessment methods used [9]. Furthermore, information about the impact of occupational exposures on disability-free survival (DFS), as opposed to mortality, in the elderly has not been reported.

Variations in exposures exist between different occupational settings, particularly during the time that the current study population was employed from 1955 to 2009 when occupational health regulations were less developed and enforced than they are today [10]. This has created challenges in establishing the impact of historical occupations in epidemiological studies. This limitation has commonly been addressed by using a Job Exposure Matrix (JEM) to relate a job title to the likely average intensity of occupational exposure at that time [11]. This allows to calculate ever and cumulative exposures across different occupations, accounting for all jobs held over the entire lifetime.

The opportunity to relate working life exposure to health in the post-retirement phase of life (including life free of disability) was provided by the ASPirin in Reducing Events in the Elderly (ASPREE) trial of low-dose aspirin versus placebo. Between 2010 and 2014, the study recruited healthy individuals aged 70 years and above whose working life was on average from 1955 to 2009 [12]. This paper aims to explore the relationship between predicted occupational exposures and pre-specified outcomes, primarily DFS, but also including all-cause and cause-specific mortality. The results allowed access to a survivor population with a wide variety of occupations, who were independently living and free of disabling chronic disease at baseline, whose health during older ages was well characterized.

## Methods

Participants in this occupational analysis were drawn from the Australian participants of the ASPREE trial which recruited healthy community-dwelling adults aged 70 years and above [12], and who also consented to enrol in the ASPREE Longitudinal Study of Older Adults (ALSOP) sub-study of ASPREE [13]. At study entry, they had no previous history of diagnosed cardiovascular disease (CVD), dementia or independence-limiting physical disability or any other chronic illness expected to limit survival to less than 5 years. Each participant was randomized to receive either daily low-dose aspirin (100 mg) or matching placebo and was followed up with annual health data collected for a median of 4.7 years [12,14,15].

A total of 12 896 ASPREE participants agreed to participate in the ALSOP sub-study which involved the completion of a medical and social questionnaire early after entry and repeated approximately 3 years after randomization. In the baseline social questionnaire, 12 498 participants provided information about their employment history by reporting up to three jobs that they had held for the longest periods during their working life. For each of these jobs, they were asked to provide job title, employer industry, description of the tasks performed and years worked [13]. Ethical approval for the ASPREE and ALSOP sub-study was obtained from the Monash University Ethics Committee (project numbers CF11/1100 and CF11/1935), and written informed consent was obtained from all participants.

A total of 36 688 job histories were coded according to the International Standard Classification of Occupations (ISCO-88) four-digit codes. The ISCO-88 job codes were then linked to the ALOHA-plus JEM which has been previously used in an Australian population [16]. To simplify the analyses, exposures to vapour, dust, gases and fumes were categorized as ‘all dust’. Similarly, those exposed to pesticides, fungicides and herbicides were combined as ‘all pesticides’ while aromatic, chlorinated and other solvents were combined and referred to as ‘all solvents’.(Table 2, available as Supplementary data at Occupational Medicine Online) The ALOHA-plus JEM classified the subjects based on job code into no, low and high exposure categories. ‘High’ in the ALOHA-plus JEM is assigned when the exposure is of high intensity and the probability of the exposure in the job is high. All other combinations of ‘high intensity’/ ‘low probability’, ‘low intensity’/ ‘high probability’, and ‘low intensity’/ ‘low probability’ are considered ‘Low’ in the ALOHA-plus JEM. For this analysis, we combined no and low exposure and defined it as never exposure and analysed the high exposure group as ‘ever high exposure’. Cumulative exposure was calculated by multiplying the ‘years worked multiplied by a particular exposure intensity’, which was then summed over the three reported longest-held jobs. The cumulative exposure was categorized into tertiles. Participants who did not report ‘years worked’ were excluded from the calculation of cumulative exposure.

DFS was defined as a composite of death, dementia and persistent physical disability, using the time to the first event, which was considered to represent a loss of capacity for independent living. The diagnosis of dementia was adjudicated according to the Diagnostic and Statistical Manual of Mental Disorders, fourth edition [15,17]. Persistent physical disability was confirmation with the loss of any one of six basic activities of daily living for a period of 6 months, or permanent admission to nursing care for physical disability if the basic daily living data could not be collected [14]. Deaths were identified during routine trial activity, and completeness of ascertainment was checked by linkage to the Australian National Death Index. After the notification of death, clinical documentation was sought from local medical practitioners, hospitals and nursing homes to allow further classification according to the underlying cause by clinical adjudicators. CVD mortality included death from myocardial infarction, other coronary heart diseases, sudden cardiac mortality, ischaemic stroke or cardiac failure [14]. Details regarding the health measures and outcome definitions were listed in Table 1 (available as Supplementary data at *Occupational Medicine* Online) and have been published previously [14].

Descriptive statistics were used for the analysis of exposure and endpoint variables. Cox proportional hazards regression models with time-to-event analysis were used to portray the relationship between occupational exposures and study endpoints. Hazard ratios (HRs) and 95% confidence intervals (CIs) were determined, and the proportional hazards assumption was validated using Schoenfeld residuals.

The data were analysed with separate strata for men and women in view of the substantial gender differences in work undertaken during the life of the ASPREE participants. A multiplicative interaction term was fitted in the adjusted Cox proportional hazards models to investigate the interaction effect between exposures with age, sex and smoking (available as Supplementary data at *Occupational Medicine* Online). Considering the correlation between exposure groups, a universal reference category was used as the reference group for all analyses (Table 2, available as Supplementary data at *Occupational Medicine* Online).

Two tiers of analysis were undertaken: in tier 1, participants with no exposures were combined with those with low exposures to form the reference group, which was then compared with the ‘ever-high exposure’ group. In tier 2, the weighted cumulative exposure in years was analysed, comparing the highest tertile with the lowest tertile. For each tier, the models were adjusted for age, sex, smoking, and education.

Sensitivity analyses were carried out using area-level socio-economic advantage and disadvantage score in quantiles as a confounder instead of education (Table 6, available as Supplementary data at *Occupational Medicine* Online). The analysis was stratified by blue- and white-collar occupations (Table 7, available as Supplementary data at *Occupational Medicine* Online), and a complete-case analysis was performed, which included all exposures from ALOHA-plus JEM (Table 8, available as Supplementary data at *Occupational Medicine* Online). We also stratified the analyses by aspirin use and placebo group (Tables 9 and 10, available as Supplementary data at *Occupational Medicine* Online) and using the same no exposure as a reference group, the analysis was presented for low and high exposure categories separately (Table 11, available as Supplementary data at *Occupational Medicine* Online). Exposure categories with less than five outcome cases were not reported for all exposure and outcome analyses. A two-sided *P* value of <0.005 was used as the cut-off for statistical significance to account for the multiplicity of analyses undertaken using Stata version 18 (StataCorp, College Station, TX).

## Results

**Figure 1.**
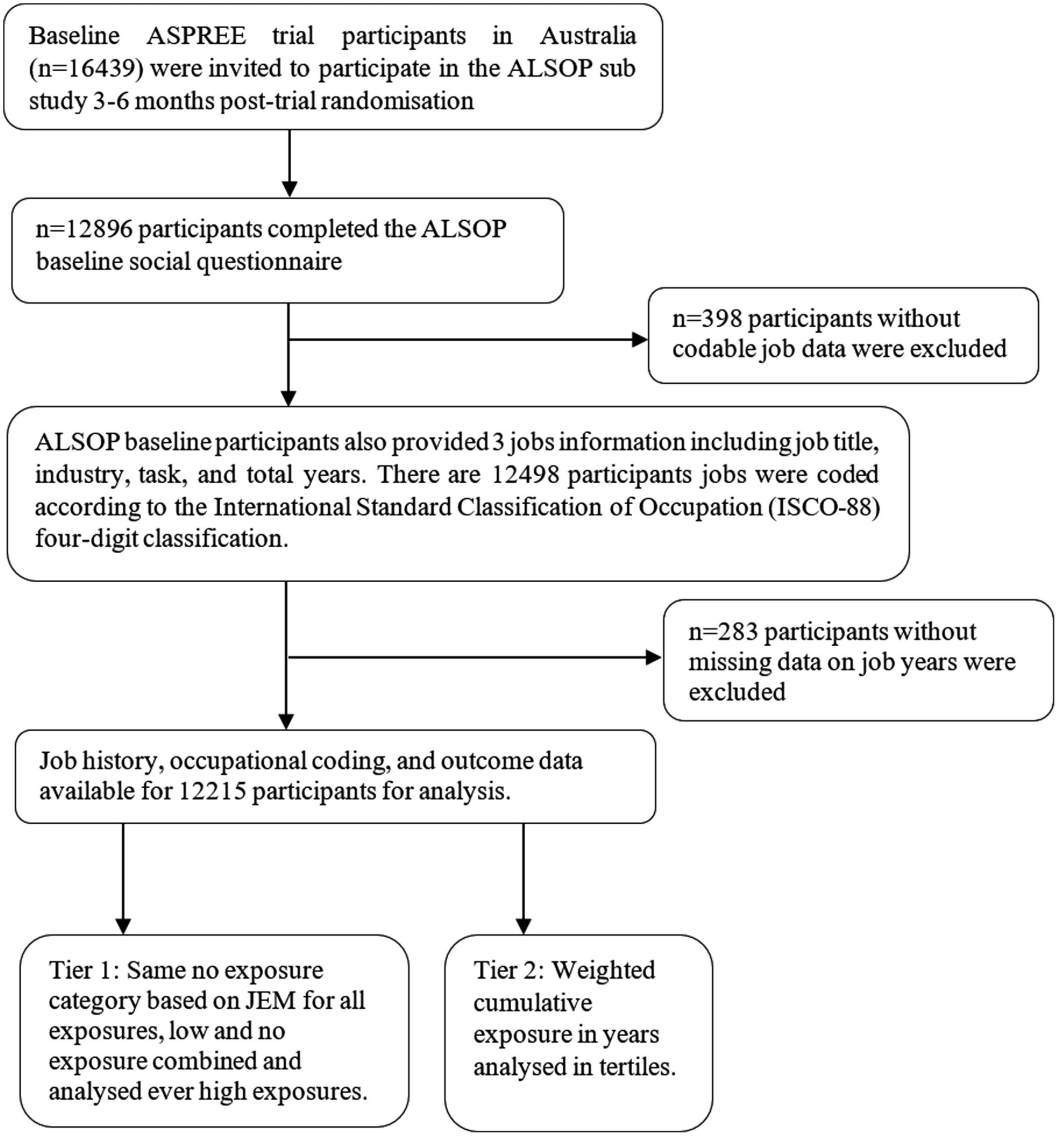
Flow chart showing the selection of participants from ALSOP cohort.

A total of 12 498 participants provided information on their three longest-held jobs, and of those, 283 participants were excluded on account of missing job titles, industry information and/or years worked leaving a total of 12 215 participants with exposure and endpoint data included in the analysis (Figure 1). Table 1 shows the baseline characteristics of the participants stratified by sex. In total, 46% (*n* = 5569) of participants were men and 54% (*n* = 6646) were women. The mean ± SD age at enrolment was 75.1 ± 4.3 years for both men and women. Most women were never (*n* = 4421, 66.5%) or former smokers (*n* = 2076, 31.2%), while men were more often former smokers (*n* = 2988, 53.6%). Amongst chronic disease risk factors, hypertension was the most prevalent in both men and women (75%), while 12% of men had diabetes (7.7 % of women) and 22% had a history of cancer.

**Table 1.** Baseline characteristics of ALSOP cohort stratified by sex (*n* = 12 215)

Study characteristics	Male, *n* (%)	Female, *n* (%)
Sex (%)	5569 (46)	6646 (54)
Age, years (mean, SD)	75.1 (4)	75.2 (4)
Living alone		
At home alone	1067 (18)	2706 (41)
At home with family, friends	4490 (81)	3920 (58)
At supervised care facility	12 (1)	18 (1)
Education (%)		
<12 years education	2498 (45)	3333 (50)
>12 years education	3017 (55)	3313 (50)
Body mass index, kg/m^2^, mean (SD)	27.9 (4)	28.0 (5)
Weight, kg, mean (SD)	83.5 (13)	71.1 (13)
Height, m, mean (SD)	1.7 (1)	1.59 (1)
Smoking history, *n* (column %)		
Never	2389 (43)	4421 (67)
Current Pack-years, median (IQR)	192 (4) 32.8 (14–55)	149 (2) 28 (17–43)
Past Pack-years, median (IQR)	2988 (54) 17 (6-32)	2076 (31) 10.8 (3-24)
Alcohol users, *n* (%)		
Never	469 (8)	1403 (21)
Current	4796 (86)	4979 (75)
Past	304 (6)	264 (4)
Waist circumference, cm, mean (SD)	101.7 (11)	92.87 (13)
Paid employment (%)		
No paid employment	46 (1)	34 (1)
Paid employment	5469 (100)	6570 (100)
Employment status (%)		
Full time	5452 (98)	5166 (80)
Part time	29 (1)	1180 (18)
Casual	12 (1)	116 (2)
Type of jobs (%)		
Blue collar	3132 (56)	2442 (37)
White collar	2437 (44)	4204 (63)
HDL cholesterol, mean (SD)	1.41 (1)	1.74 (1)
LDL cholesterol, mean (SD)	3.02 (1)	3.12 (1)
Frailty (%)		
Non frail	3592 (65)	4188 (63)
Pre frail	1903 (34)	2347 (35)
Frail	74 (1)	111 (2)
Chronic kidney disease (%)	1233 (24)	1592 (26)
Diabetes (%)	644 (12)	514 (8)
Hypertension (%)	4187 (75)	4871 (73)
Cancer history (%)	1239 (22)	1152 (17)

**Figure 2.**
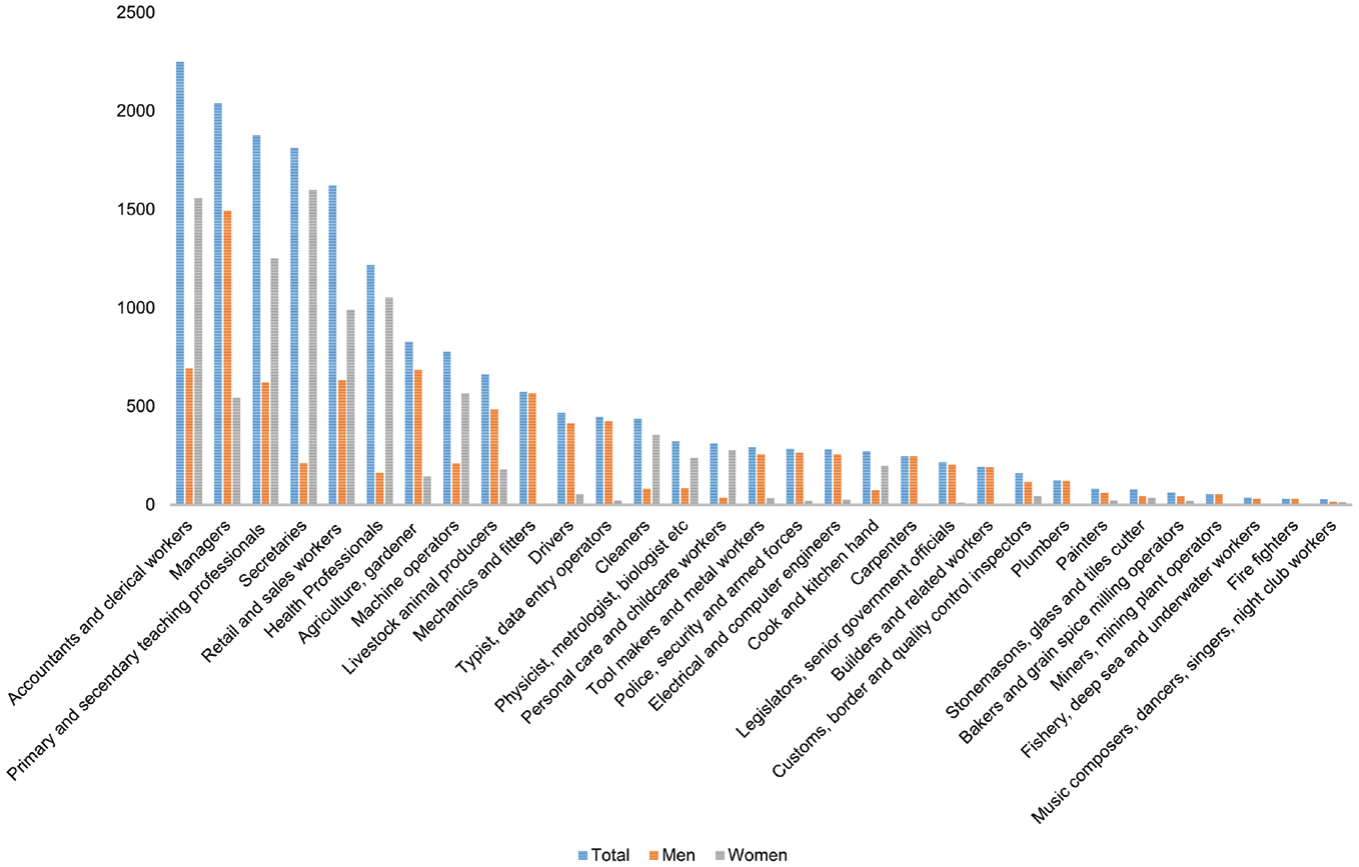
Prevalence of common occupations in ALSOP cohort.

Figure 2 shows the most commonly reported occupations across the cohort. Amongst men, 43.8% had worked predominantly in ‘white-collar” occupations and 56.2% in ‘blue-collar” occupations, whereas in women, the corresponding percentage were 63.3% and 36.7%, respectively. The top five most commonly reported occupations amongst men were managerial (*n* = 1494), accountancy (*n* = 693), agriculture (*n* = 685), retail (*n* = 632) and mechanics and fitters (*n* = 624). Amongst women the most commonly reported occupations were secretarial (*n* = 1600), accountancy (*n* = 1559), teaching (*n* = 1253), health professions (*n* = 1054) and retail (*n* = 991) (Table 3, available as Supplementary data at *Occupational Medicine* Online).

Substantial numbers of participants reported working in occupations that were exposed to dust (*n* = 1948), solvents (*n* = 621), metals (*n* = 473) and pesticides (*n* = 274) (Figure 1, available as Supplementary data at *Occupational Medicine* Online). Most pesticide exposure resulted from work in agriculture, livestock animal production, and gardening or landscaping. Those most likely to be associated with all-dust exposure were mechanics, fitters, machine operators and tool makers, whereas all solvent exposure was most likely amongst mechanics, fitters and painters (Table 4, available as Supplementary data at *Occupational Medicine* Online).

Table 2 demonstrates that amongst both sexes, both ‘ever high’ and upper tertiles of cumulative exposure to all dust (HR 1.24; 95% CI 1.07–1.43, *P* < 0.005 for ever high and 1.23; 1.09–1.43, *P* < 0.005 for cumulative exposure) and all pesticides (1.67; 1.21–2.31, *P* < 0.005 for ever high and 1.66; 1.17–2.33, *P* < 0.005 for cumulative exposure) were associated with a significant reduction in DFS compared with those never exposed. The association with high dust exposure was primarily driven by biological dust exposure, and high herbicides and insecticides groups influenced high pesticides, as we observed in the complete-case analysis presented in Table 8 (available as Supplementary data at *Occupational Medicine* Online). No association was observed for all-solvent and all-metal exposures.

**Table 2.** Associations for ever-high and cumulative exposures to all dust, all pesticides, all solvents, and all metals and ASPREE endpoints in ALSOP cohort

Occupational exposures	Disability-free survival			All-cause mortality			Cancer-related mortality			CVD-related mortality		
	*n* ^*^	HR (95% CI)	*P* value	*n*	HR (95% CI)	*P* value	*n*	HR (95% CI)	*P* value	n	HR (95% CI)	*P* value
***High all-dust exposures*** ^a^	311	**1.24 (1.07–1.43)**	**0.004**	213	1.14 (0.96–1.36)	0.125	100	1.12 (0.88–1.42)	0.369	53	1.01 (0.71–1.42)	0.971
Cumulative exposures in upper tertile^b^	277	**1.23 (1.09–1.43)**	**0.002**	189	1.14 (0.94–1.38)	0.18	94	1.05 (0.81–1.36)	0.691	46	1.00 (0.67–1.49)	0.991
***High all pesticide exposure***	43	**1.67 (1.21–2.31)**	**0.002**	30	1.30 (0.88–1.94)	0.189	13	1.36 (0.83–2.23)	0.216	5	0.85 (0.46–1.57)	0.615
Cumulative exposures in upper tertile	31	**1.66 (1.17–2.33)**	**0.004**	19	**1.69 (1.14–2.74)**	**0.033**	10	1.32 (0.69–2.48)	0.369	–	–	–
***High all solvents exposure***	108	1.15 (0.96–1.38)	0.122	60	**1.41 (1.10–1.79)**	**0.006**	28	1.32 (0.94–1.83)	0.106	18	**1.59 (1.01–2.55)**	**0.048**
Cumulative exposures in upper tertile	133	1.15 (0.93–1.25)	0.327	85	1.01 (0.83–1.24)	0.865	43	0.92 (0.68–1.23)	0.563	23	1.17 (0.79–1.72)	0.436
***High all metals exposure***	83	**1.29 (1.04–1.61)**	**0.023**	50	**1.38 (1.04–1.63)**	**0.025**	21	1.25 (0.82–1.90)	0.284	17	**1.64 (1.01–2.64)**	**0.044**
Cumulative exposures in upper tertile	73	1.15 (0.92–1.44)	0.218	44	1.19 (0.90–1.58)	0.219	18	1.19 (0.77–1.86)	0.426	15	1.29 (0.82–2.04)	0.261

The model was adjusted for age, sex, smoking and education. Data are shown for cancer-related mortality that were related to primary or metastatic cancer and adverse effect from cancer treatment. Cardiovascular (CVD) mortality was defined as all-cause ischaemic event (myocardial infarction, other coronary heart disease, sudden cardiac mortality, or ischaemic stroke).

^a^Participants with no and low exposure were combined and used as a reference category.

^b^Participants without years of exposure (zero years) were used as a reference category.

^*^ *n* = number people with exposure and endpoints.

^**^Bold are statistically significant, if *P* < 0.005 and *P* < 0.05.

Tables 3 and 4 show a similar analysis stratified by sex. In men, ‘ever high’ exposure to all dust (1.25; 1.07–1.46, *P* < 0.005 for ever high and 1.25; 1.05–1.44, *P* < 0.005 for cumulative exposure) and all pesticides (1.64; 1.16–2.29, *P* < 0.005 for ever high and 1.61; 1.06–2.43, *P* < 0.005 for cumulative exposure) were similarly associated with reduced DFS, whereas in women, there was no association for high dust and high solvent exposure. DFS was reduced amongst participants whose occupation involved high exposure to all dust and pesticides.

**Table 3.** Association for ever-high and cumulative exposures to all dust, all pesticides, all solvents and all metals and ASPREE endpoints in men in ALSOP Cohort

Occupational exposures	Disability-free survival			All-cause mortality			Cancer-related mortality			CVD-related mortality		
	*n* ^*^	HR (95% CI)	*P* value	*n*	HR (95% CI)	*P* value	*n*	HR (95% CI)	*P* value	N	HR (95% CI)	*P* value
***High all-dust exposures*** ^a^	285	**1.25 (1.07–1.46)**	**0.005**	195	1.10 (0.92–1.33)	0.296	94	1.10 (0.85–1.41)	0.482	48	0.96 (0.66–1.39)	0.832
Cumulative exposures in upper tertile^b^	266	**1.25 (1.05–1.44)**	**0.012**	181	1.12 (0.91–1.38)	0.267	89	1.07 (0.81–1.41)	0.615	45	0.88 (0.57–1.34)	0.538
***High all pesticide exposure***	39	**1.64 (1.16–2.29)**	**0.004**	26	1.35 (0.86–2.12)	0.18	12	**1.51 (1.02–2.23)**	**0.039**	–	–	–
Cumulative exposures in upper tertile	30	**1.61 (1.06–2.43)**	**0.004**	19	**1.72 (1.05–2.80)**	**0.003**	10	**2.39 (1.18–4.85)**	**0.016**	–	–	–
***High all solvents exposure***	91	**1.21 (1.50–1.48)**	**0.029**	52	**1.43 (1.17–1.81)**	**0.004**	26	1.38 (0.94–2.02)	0.10	15	1.28 (0.81–2.05)	0.292
Cumulative exposures in upper tertile	110	1.09 (0.92–1.29)	0.275	73	1.01 (0.8–1.26)	0.947	38	0.93 (0.67–1.31)	0.69	20	1.17 (0.77–1.77)	0.461
***High all metals exposure***	82	**1.29 (1.03–1.59)**	**0.023**	50	**1.36 (1.01–1.84)**	**0.038**	28	1.31 (0.8–1.98)	0.314	17	1.51 (0.95–2.39)	0.083
Cumulative exposures in upper tertile	73	1.15 (0.93–1.43)	0.202	44	1.18 (0.88–1.58)	0.257	18	1.19 (0.75–1.89)	0.462	15	1.21 (0.77–1.91)	0.396

The model was adjusted for age, smoking and education. Data are shown for cancer-related mortality that were related to primary or metastatic cancer and adverse effect from cancer treatment. Cardiovascular (CVD) mortality was defined as all-cause ischaemic event (myocardial infarction, other coronary heart disease, sudden cardiac mortality, or ischaemic stroke).

^a^Participants not exposed to any exposure and low exposure to particular exposure were combined and used as a reference category.

^b^Participants without years of exposure (zero years) were used as a reference category.

^*^ *n* = number people with exposure and endpoints.

^**^Bold are statistically significant, if *P* < 0.005 and *P* < 0.05.

**Table 4.** Associations for ever-high and cumulative exposures to all dust, all pesticides, all solvents and all metals and ASPREE endpoints in women in ALSOP cohort

Occupational exposures	Disability-free survival			All-cause mortality			Cancer-related mortality			CVD-related mortality		
	*n* ^*^	HR (95% CI)	*P* value	*n*	HR (95% CI)	*P* value	*n*	HR (95% CI)	*P* value	*n*	HR (95% CI)	*P* value
***High all dust exposures*** ^a^	26	1.13 (0.74–1.75)	0.567	18	**1.64 (1.16–2.31)**	**0.003**	6	**1.96 (1.24–3.10)**	**0.004**	5	1.47 (0.65–3.31)	0.349
Cumulative exposures in upper tertile^b^	11	1.32 (0.72–2.39)	0.368	8	**1.93 (1.29–2.86)**	**0.001**	5	**1.91 (1.08–3.37)**	**0.026**	–	–	–
***High all solvents exposure***	17	0.89 (0.59–1.35)	0.59	8	1.27 (0.81–1.99)	0.428	–	–	–	–	–	**–**
Cumulative exposures in upper tertile	23	1.06 (0.79–1.45)	0.671	12	1.06 (0.69–1.64)	0.79	5	0.92 (0.53–1.61)	0.779	–	–	–

The model was adjusted for age, smoking and education. Data are shown for cancer-related mortality that were related to primary or metastatic cancer and adverse effect from cancer treatment. Cardiovascular (CVD) mortality was defined as All-cause ischaemic event (myocardial infarction, other coronary heart disease, sudden cardiac mortality, or ischaemic stroke). Less than five cases for exposure to pesticides and metals were not reported.

^a^Participants not exposed to any exposure and low exposure to particular exposure were combined and used as a reference category.

^b^Participants without years of exposure (zero years) were used as a reference category.

^*^ *n* = number people with exposure and endpoints.

^**^Bold are statistically significant, if *P* < 0.005 and *P* < 0.05.

Amongst men, those in the upper tertile of cumulative all pesticide exposure (1.72; 1.05–2.8, *P* < 0.005) and those with ‘ever-high’ exposure to all solvents (1.43; 1.17–1.81, *P* < 0.005) were found to have an increase in all-cause mortality (*P*-trend < 0.005) (Table 3). Amongst women, ever-high (1.64; 1.16–2.31, *P* < 0.005) and cumulative exposure (1.93; 1.29–2.86, *P* < 0.005) to all dusts was associated with increased all-cause mortality, where the events number was very low (Table 4). These relationships remained after adjustment for co-exposure to other occupational exposures (data not shown) and were independent of aspirin use in this study population (Tables 9 and 10, available as Supplementary data at *Occupational Medicine* Online).

Amongst men, high pesticides and upper tertiles of cumulative exposure were associated with increased cancer-related mortality but neither exposure to all dusts, all pesticides, all solvents and all metals were associated with increased CVD-related mortality (Table 3). Amongst women, ‘ever high’ exposure to all dust was associated with increased cancer-related mortality (Table 4).

## Discussion

This survivor cohort was predominantly employed as white-collar workers. The number of individuals with specific occupational exposures were small. However, when exposures to different dusts, solvents, pesticides and metals were combined, the number of exposed individuals were sufficient to identify a significant difference in DFS or increased risk of all-cause, cancer or CVD mortality.

The principal finding was that after controlling for differences in education level and smoking habit, both ‘ever high’ and cumulative exposure to all dusts and all pesticides during a person’s working years were associated with reduced DFS after age 70. This was not seen with all-solvent or all-metal exposure, suggesting that exposure to all dusts and pesticides may be associated with specific risks. All-cause mortality was increased amongst men exposed to all solvents, while in women, higher exposure to all dusts (predominantly biological dust) was associated with an increased risk of all-cause and cancer-related mortality.

No previous longitudinal study has examined the relationship in a population sample between earlier occupational exposure to dust and DFS in the post-retirement period. DFS is a measure of a person’s ability to live independently and therefore indicates a better quality of life and less economic burden to the community. Identifying occupational exposures that adversely affect this outcome may lead to new prevention strategies. In previous studies, a variety of occupational exposures have been reported to influence other aspects of health including mortality at older ages [18,19]. It is, therefore, expected that DFS could be influenced in a similar manner.

These results extend the findings from earlier community-based studies looking at the relationship between occupational exposures and long-term health outcomes summarized in Table 5 (available as Supplementary data at *Occupational Medicine* Online). Most have focused on mortality but with inconsistent results. The Dutch Ageing Cohort Study using self-reported exposure found no difference in life expectancy amongst individuals [19], whereas, like this study, a report from the Honolulu Heart Program (HHP) found that several occupational exposures including dusts, pesticides and solvents, were associated with reduced survival [18]. However, mortality is a crude measure of health in older age because it does not capture time spent with serious physical or cognitive disability.

Previous studies have also reported long-term adverse health effects from exposure to dusts and pesticides. A longitudinal study from an industrial town in the Netherlands with participants aged 40-–9 years found an increase in mortality, specifically amongst those exposed to dust rather than pesticides, solvents or metals [20]. Dust particles, which vary in size, shape and composition, may damage DNA and cause immunosuppression leading to airway irritation and heart disease [21]. These results emphasize the importance of monitoring and mitigating dust exposure in occupational settings with the potential benefit of better health in the years after retirement.

The finding that all pesticide exposure was also associated with reduced DFS is plausible considering reports linking pesticide exposure to forms of chronic morbidity including cancer, chronic respiratory disease and depression [22]. The participants exposed to all pesticides included those working as farm hands, crop growers, foresters, gardeners and landscape workers. Likely agents included organochlorine dichlorodiphenyltrichloroethane (DDT), aldrin, dieldrin, parathion, paraquat and similar compounds. During the early years of the working life of the ASPREE participants, regulations governing all pesticide exposure were typically less stringent and a variety of long-acting agents, for example, DDT, which could accumulate in body fat were in common use.

The strengths of this study included the rigorous identification of the composite endpoint (deaths, dementia and physical disability) through annual contact with participants and access to medical records with minimal loss to follow-up [15]. Similarly, the cause of death was determined by access to clinical records and death registry linkage rather than reliance on ICD codes. The use of the JEM allowed participants to just recall their principal occupations rather than specific exposures, thus reducing the likelihood of recall bias for specific exposures. The relatively larger sample size included a broad spectrum of occupations and the data allowed adjustment for a range of confounders, including smoking and education.

The study also had certain limitations. Participants reported their three longest-held jobs, but the information provided may have been influenced by a selective recall bias. The predicted exposures obtained using a JEM are a prediction of the true exposures. The study only includes individuals who have survived into a post-retirement phase so individuals who retired early, developed ill health or died from occupational diseases are not reflected in the results. The fewer identified hazards amongst women are likely to reflect their generally shorter time in the workplace and smaller numbers with significant exposures. Additionally, the participants volunteering for the study were relatively healthy at entry. However, the focus of the study was to identify occupational factors impairing health in older age and the broad range of occupations amongst the large cohort allowed a contrast in outcomes amongst different occupational groups that could not be gathered from most other epidemiological studies. The analyses combined participants randomized to both aspirin and placebo. This was justified because aspirin use is (and has long been) common in the community, and it had no effect on the incidence of DFS. However, because of the small effect of aspirin increasing mortality in the ASPREE study, we undertook two sensitivity analyses, the first of which showed that adjusting for aspirin use as an additional confounder made no difference to the overall effect estimates. Secondly, a stratified analysis presenting the analyses separately by aspirin/placebo (Tables 9 and Table 10, available as Supplementary data at *Occupational Medicine* Online) also showed no difference.

In conclusion, this community-based study, with rigorous follow-up of health outcomes, demonstrated that specific exposures occurring during one’s working life may affect DFS in the post-retirement years. Decreasing harmful exposure to all dusts, all solvents and all pesticides through personal protective equipment and engineering controls could reduce the late-life burden of morbidity amongst workers who regularly use these agents.

## Supplementary Material

kqad105_suppl_Supplementary_Material
