# Supplementary material for: Long-term occupational exposures on disability-free survival and mortality in older adults
Source: Occup Med (Lond). 2023 Nov 8;73(8):492–9. doi: 10.1093/occmed/kqad105 (PMC10756660; doi:10.1093/occmed/kqad105)
Supplement: kqad105_suppl_Supplementary_Material [file kqad105_suppl_supplementary_material.docx]

Impact of Long-term occupational exposures on disability-free survival and mortality in older adults

# Online supplementary

## Methods

Based on Spearman’s correlation map, there was a significant overlap between subgroups of dust, pesticide and solvent exposures (online supplemental **Table S 1**). Furthermore, sparse data for the subgroups of pesticides made it impossible to disentangle their specific effects. For these reasons, we used a combined variable, vapor, dust, gases and fumes, all pesticides in the analyses, rather than biological dust, mineral dust, gases/fumes, herbicides, insecticides, fungicides individually, and all solvents were combined, instead of using aromatic and chlorinated solvents.

## Covariates

The potential confounders were identified using DAGitty software, which suggested age, sex, smoking, and education as covariates. Participants' lifetime smoking status was categorized as either ever or never-smokers. In the sensitivity analysis, the confounding variable was changed from education to the index of relative socioeconomic advantage and disadvantage score in quantiles, as presented in Table S6, of the primary analysis presented in Table 2.

**Table S 1** ASPREE health measures and definitions

| ***Annual health measurements*** |
| --- |
| - Demographic and lifestyle factors |
| - Weight, height, smoking status-current, former, never |
| - Depression screening questionnaire |
| - LIFE disability questionnaire including the Katz basic Activities of Daily Living (including walking across a room, bathing, dressing, transferring from a bed or chair, using the toilet, and eating); participants selected one of the following options for completing these tasks with ‘no difficulty’, ‘a little difficulty’ ‘some difficulty’, ‘a lot of difficulty’ or ‘unable to perform independently’; and, as a check, answered whether assistance from another person was required to complete |
| - Other clinical events |
| ***6-month phone calls*** |
| - confirmation of living circumstances |
| - administration of the Katz basic Activities of Daily Living |
| - clinical and adverse events report |
| ***Biennial health measures*** |
| - neurocognitive assessments |
| - physical function tests |

**Table S 2** Spearman rank correlation map between exposures to dust, pesticides, solvents and metals

| **Rank correlation heat map** | **Dusts** | | | | **Pesticides** | | | | **Solvents** | | | |
| --- | --- | --- | --- | --- | --- | --- | --- | --- | --- | --- | --- | --- |
|  | ***Biological Dust*** | ***Mineral Dust*** | ***Gases and Fumes*** | ***VGDF*** | ***All pesticides*** | ***Herbicides*** | ***Insecticides*** | ***Fungicides*** | ***Aromatic solvents*** | ***Chlorinated solvents*** | ***Other solvents*** | ***Metals*** |
| ***Biological Dust*** | 1.00 |  |  |  |  |  |  |  |  |  |  |  |
| ***Mineral Dust*** | 0.45 | 1.00 |  |  |  |  |  |  |  |  |  |  |
| ***Gases and Fumes*** | 0.55 | 0.68 | 1.00 |  |  |  |  |  |  |  |  |  |
| ***VGDF*** | 0.72 | 0.72 | 0.89 | 1.00 |  |  |  |  |  |  |  |  |
| ***All pesticides*** | 0.51 | 0.52 | 0.32 | 0.45 | 1.00 |  |  |  |  |  |  |  |
| ***Herbicides*** | 0.36 | 0.43 | 0.26 | 0.33 | 0.76 | 1.00 |  |  |  |  |  |  |
| ***Insecticides*** | 0.5 | 0.51 | 0.3 | 0.43 | 0.95 | 0.75 | 1.00 |  |  |  |  |  |
| ***Fungicides*** | 0.48 | 0.5 | 0.3 | 0.42 | 0.96 | 0.74 | 0.93 | 1.00 |  |  |  |  |
| ***Aromatic solvents*** | 0.26 | 0.5 | 0.56 | 0.55 | 0.24 | 0.3 | 0.23 | 0.24 | 1.00 |  |  |  |
| ***Chlorinated solvents*** | 0.05 | 0.39 | 0.51 | 0.43 | -0.02 | -0.02 | -0.04 | -0.03 | 0.71 | 1.00 |  |  |
| ***Other solvents*** | 0.34 | 0.21 | 0.59 | 0.53 | -0.03 | -0.04 | -0.05 | -0.05 | 0.58 | 0.59 | 1.00 |  |
| ***Metals*** | -0.01 | 0.46 | 0.46 | 0.4 | 0.01 | -0.004 | -0.02 | 0.01 | 0.54 | 0.69 | 0.45 | 1.00 |


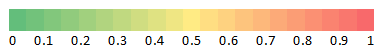


Colour-coding (-1 to 0 not shown):

**Figure S 1** Number of exposures in the cohort

**Table S 3** Crude numbers of common occupations among men and women in the cohort

| **Common occupations in the cohort** | **Number of reported jobs** | **Men** | **Women** |
| --- | --- | --- | --- |
| *Accountants and clerical workers* | 2252 | 693 | 1559 |
| *Mangers* | 2039 | 1494 | 545 |
| *Primary and secondary teaching professionals* | 1877 | 624 | 1253 |
| *Secretaries* | 1812 | 212 | 1600 |
| *Retail and sales workers* | 1623 | 632 | 991 |
| *Health Professionals* | 1218 | 164 | 1054 |
| *Agriculture, gardener* | 829 | 685 | 144 |
| *Machine operators* | 778 | 210 | 568 |
| *Livestock animal producers* | 664 | 484 | 180 |
| *Mechanics and fitters* | 573 | 568 | 5 |
| *Drivers* | 468 | 414 | 54 |
| *Typist, data entry operators* | 448 | 425 | 23 |
| *Cleaners* | 437 | 82 | 355 |
| *Physicist, metrologist, biologist etc* | 323 | 83 | 240 |
| *Personal care and childcare workers* | 314 | 37 | 277 |
| *Tool makers and metal workers* | 292 | 258 | 34 |
| *Police, security and armed forces* | 285 | 264 | 20 |
| *Electrical and computer engineers* | 284 | 257 | 27 |
| *Cook and kitchen hand* | 272 | 73 | 199 |
| *Carpenters* | 247 | 246 | 1 |
| *Legislators, senior government officials* | 216 | 204 | 12 |
| *Builders and related workers* | 194 | 192 | 2 |
| *Customs, border and quality control inspectors* | 160 | 117 | 43 |
| *Plumbers* | 124 | 123 | 1 |
| *Painters* | 81 | 60 | 21 |
| *Stonemasons, glass and tiles cutter* | 79 | 43 | 36 |
| *Bakers and grain spice milling operators* | 62 | 43 | 19 |
| *Miners, mining plant operators* | 55 | 54 | 1 |
| *Fishery, deep sea and underwater workers* | 37 | 30 | 7 |
| *Fire fighters* | 30 | 30 | 0 |
| *Music composers, dancers, singers, night club workers* | 28 | 15 | 13 |

**Table S4** Crude numbers and exposures in the cohort

| ***Main occupations*** | ***Exposures*** | ***Numbers*** |
| --- | --- | --- |
| Agriculture/Gardeners/landscaping | All pesticides | 269 |
| Livestock animal producers | All pesticides | 163 |
| Mechanics/fitters/machine operators/tool makers | All dust | 752 |
| Agriculture/Gardeners/landscaping | All dust | 564 |
| Livestock animal producers | All dust | 450 |
| Mechanics/fitters/tool makers | All solvents | 508 |
| Painters | All solvents | 143 |

**Table S5** Population-based cohort studies which investigated occupational exposures and all-cause and cause-specific mortality

| **Author, year** | **Country and cohort** | **Sample size** | **Duration of follow-up (years)** | **Age of cohort (years)** | **Method of exposure assessment** | **Outcome data source** | **Summary of the studies** | **Confounders adjusted** |
| --- | --- | --- | --- | --- | --- | --- | --- | --- |
| **Disability and mortality** | | | | | | | | |
| de Wind et al. 2020^21^ | The Netherlands. LASA | 2513 | 24 | 55-85 | GPJEM-longest job held | Disability from questionnaire and mortality from population register | Lower physical demands, higher psychological work demands, greater psychological work resources to live more disability free years. No difference of life expectancy with occupational exposures. | Age and sex, age was included as a time-varying covariates. |
| **All-cause mortality** | | | | | | | | |
| Niedhammer, I. et al. 2021^25^ | France, STRESSJEM data linkage cohort | 1496332 | Data linkages from 1976-2002 | 27 | STRESSJEM | French National death registry | Psychological work exposures increase the risk of all-cause mortality | Age as time scale variable, calendar time, biomechanical, physical, chemical and biological exposure were adjusted. |
| Niedhammer, I. et al. 2022^26^ | France, STRESSJEM data linkage cohort | 1496332 | Data linkages from 1976-2002 | 27 | STRESSJEM | French National death registry | Shift work increase the risk of all-cause, cancer and other mortality | Age as time scale variable, calendar time, biomechanical, physical, chemical and biological exposure were adjusted. |
| Tanaka et al. 2020^27^ | Japan | 24863422 | 5 | 25-64 | National Census Data | NVS and MHLW | High all-cause and cardiovascular mortality in agriculture, forestry, fishing, mining, manufacturing, health and transport workers | Age-standardised mortality for men |
| **Cancer-related mortality** | | | | | | | | |
| Eguchi et al. 2017^28^ | Japan, National health survey data | 27333396 | 5 | 25-64, only men | Occupational data from ministry of labour | 2010 National population census data | Occupations such as agriculture, forestry, fisheries, professionals and engineering had higher mortality of lung, gastric and colorectal cancer. | Age, sex, industry |
| **Cardiovascular mortality** | | | | | | | | |
| Charles et al. 2010^20^ | USA, HHP | 7540 | 3 | <58 | Self-reported questionnaire and expert assessment | Surveillance system, hospital records, and death certificates. | Occupational exposure to pesticides, metals, and solvents obtained during middle age was independently associated with increased mortality from all causes, cancer, and in specific cases, circulatory diseases. | Education, smoking status, triglycerides, physical activity, alcohol intake, and systolic blood pressure. |

GPJEM=General Population Job Exposure Matrix; LASA=Longitudinal Ageing Study Amsterdam;MHLW=Ministry of Health, Labour and Welfare; NDI=National Death Index; NVS=National Vital Statistics

Neidhammer et al. study linked two data sources, job history from 1976-2002 from French National working population of employees and French National

**Table S6** Associations for ever high and cumulative exposures to dust, pesticides, solvents and metals and ASPREE endpoints; sensitivity analysis adjusted for socio-economic index instead of education in the final model

| **Occupational exposures** | **Disability-free survival** | | | **All-cause mortality** | | | **Cancer-related mortality** | | | **CVD related mortality** | | |
| --- | --- | --- | --- | --- | --- | --- | --- | --- | --- | --- | --- | --- |
|  | **n*** | **HR (95% CI)** | **p-value** | **n** | **HR (95% CI)** | **p-value** | **n** | **HR (95% CI)** | **p-value** | **n** | **HR (95% CI)** | **p-value** |
| ***High all dust exposures*** † | 311 | **1.19 (1.03-1.37)** | **0.013** | 213 | 1.11 (0.94-1.31) | 0.213 | 100 | 1.11 (0.87-1.40) | 0.406 | 53 | 1.06 (0.75-1.50) | 0.742 |
| Cumulative exposures in upper tertile‡ | 277 | **1.18 (1.01-1.38)** | **0.035** | 189 | 1.11 (0.92-1.34) | 0.26 | 94 | 1.04 (0.80-1.35) | 0.731 | 46 | 1.07 (0.72-1.61) | 0.718 |
|  | | | | | | | | | | | | |
| ***High all pesticide exposure*** | 43 | **1.52 (1.08-2.14)** | **0.015** | 30 | 1.24 (0.83-1.88) | 0.288 | 13 | 1.32 (0.81-2.18) | 0.275 | 5 | 0.92 (0.50-1.67) | 0.779 |
| Cumulative exposures in upper tertile | 31 | **1.61 (1.14-2.26)** | **0.006** | 19 | **1.66 (1.02-2.69)** | **0.038** | 10 | 1.32 (0.69-2.50) | 0.389 | - | - | - |
|  | | | | | | | | | | | | |
| ***High all solvents exposure*** | 108 | 1.12 (0.93-1.36) | 0.219 | 60 | **1.40 (1.11-1.77)** | **0.005** | 28 | 1.35 (0.97-1.88) | 0.073 | 18 | **1.67 (1.04-2.70)** | **0.033** |
| Cumulative exposures in upper tertile | 133 | 1.07 (0.92-1.25) | 0.339 | 85 | 1.03 (0.84-1.25) | 0.807 | 43 | 0.92 (0.68-1.25) | 0.612 | 23 | 1.17 (0.79-1.75) | 0.414 |
|  | | | | | | | | | | | | |
| ***High all metals exposure*** | 83 | **1.25 (1.00-1.57)** | **0.049** | 50 | **1.36 (1.03-1.78)** | **0.028** | 21 | 1.29 (0.86-1.93) | 0.224 | 17 | **1.69 (1.05-2.73)** | **0.032** |
| Cumulative exposures in upper tertile | 73 | 1.13 (0.90-1.43) | 0.278 | 44 | 1.19 (0.90-1.58) | 0.21 | 18 | 1.23 (0.79-1.90) | 0.361 | 15 | 1.35 (0.84-2.17) | 0.212 |

The model was adjusted for age, sex, smoking and socioeconomic index

†Participants not exposed to any exposure and low exposure to particular exposure were combined and used as a reference category.

‡Participants without years of exposure (zero years) were used as a reference category.

a) Data are shown for cancer-related mortality that were related to primary or metastatic cancer and adverse effect from cancer treatment.

b) Cardiovascular (CVD) mortality was defined as all-cause ischemic event (myocardial infarction, other coronary heart disease, sudden cardiac mortality, or ischemic stroke).

*n=number people with exposure and endpoints

**Bold are statistically significant, if p<0.005 and p<0.05

| **Occupational exposures** | **Disability free survival** | | | **All-cause mortality** | | | **Cancer-related mortality** | | | **CVD related mortality** | | | |
| --- | --- | --- | --- | --- | --- | --- | --- | --- | --- | --- | --- | --- | --- |
|  | **n*** | **HR (95% CI)** | **p-value** | **n** | **HR (95% CI)** | **p-value** | **n** | **HR (95% CI)** | **p-value** | **N** | **HR (95% CI)** | **p-value** | |
| ***High all dust exposures*** |  |  |  |  |  |  |  |  |  |  |  |  | |
| *Blue collar* | 305 | **1.19 (1.01-1.42)** | **0.042** | 208 | 1.11 (0.89-1.38) | 0.332 | 98 | 1.06 (0.79-1.43) | 0.704 | 53 | 1.07 (0.72-1.6) | 0.722 | |
| *White collar* | 6 | 1.78 (0.98-3.22) | 0.055 | 5 | 1.87 (0.92-3.8) | 0.082 | 2 | 1.93 (0.96-1.00) | 0.167 | 0 | - | - | |
|  | | | | | | | | | | | | | |
| ***High all pesticide exposure*** |  |  |  |  |  |  |  |  |  |  |  |  | |
| *Blue collar* | 43 | **1.57 (1.12-2.21)** | **0.004** | 30 | 1.21 (0.82-1.78) | 0.340 | 13 | 1.23 (0.71-2.15) | 0.46 | 5 | 0.92 (0.42-1.96) | 0.82 | |
| *White collar* | 0 | **-** | **-** | 0 | **-** | **-** | 0 | **-** | **-** | 0 | - | - | |
|  | | | | | | | | | | | | |  |
| ***High all solvents exposure*** |  |  |  |  |  |  |  |  |  |  |  |  | |
| *Blue collar* | 108 | 1.1 (0.89-1.34) | 0.362 | 60 | **1.41 (1.1-1.81)** | **0.003** | 28 | **1.43 (1.01-2.07)** | **0.045** | 18 | 1.59 (0.9-2.8) | 0.11 | |
| *White collar* | 0 | - | - | 0 | - | - | 0 | - | - | 0 | - | - | |
|  | | | | | | | | | | | | | |
| ***High all metals exposure*** |  |  |  |  |  |  |  |  |  |  |  |  | |
| *Blue collar* | 83 | 1.22 (0.94-1.59) | 0.141 | 50 | 1.32 (0.96-1.82) | 0.096 | 21 | 1.28 (0.79-2.08) | 0.321 | 17 | 1.59 (0.84-3.04) | 0.155 | |
| *White collar* | 0 | - | - | 0 | - | - | 0 | - | - | 0 | - | - | |

**Table S7** Association between ever high exposures to dust, pesticides, solvents and metals and ASPREE endpoints in blue- and white-collar occupations in ALSOP cohort

The model was adjusted for age, sex, smoking and education

**Table S8** Associations for ever high exposures to all ALOHA plus JEM exposure categories and ASPREE endpoints (complete-case analysis)

| **Occupational exposures** | **Disability free survival** | | | **All-cause mortality** | | | **Cancer-related mortality** | | | **CVD related mortality** | | |
| --- | --- | --- | --- | --- | --- | --- | --- | --- | --- | --- | --- | --- |
|  | **n*** | **HR (95% CI)** | **p-value** | **n** | **HR (95% CI)** | **p-value** | **n** | **HR (95% CI)** | **p-value** | **n** | **HR (95% CI)** | **p-value** |
| ***High biological dust*** | 465 | 0.99 (0.88-1.11) | 0.852 | 282 | 1.01 (0.87-1.17) | 0.91 | 141 | 0.9 (0.74-1.11) | 0.336 | 60 | 0.95 (0.69-1.32) | 0.779 |
| *Men* | 217 | **1.22 (1.02-1.45)** | **0.033** | 151 | 1.05 (0.85-1.31) | 0.63 | 77 | 1.03 (0.77-1.37) | 0.856 | 27 | 0.77 (0.47-1.26) | 0.298 |
| *Women* | 248 | **0.84 (0.72-0.98)** | **0.03** | 131 | 0.97 (0.79-1.2) | 0.81 | 64 | 0.82 (0.61-1.1) | 0.19 | 33 | 1.13 (0.73-1.74) | 0.579 |
| ***High mineral dust*** | 342 | 1.12 (0.97-1.3) | 0.119 | 234 | 1.03 (0.87-1.24) | 0.669 | 107 | 0.88 (0.68-1.13) | 0.315 | 62 | 1.02 (0.74-1.42) | 0.889 |
| *Men* | 289 | **1.22 (1.03-1.44)** | **0.019** | 197 | 1.13 (0.93-1.36) | 0.218 | 93 | 1.06 (0.82-1.38) | 0.649 | 49 | 0.85 (0.58-1.25) | 0.401 |
| *Women* | 53 | 0.91 (0.68-1.23) | 0.56 | 37 | 0.85 (0.59-1.24) | 0.41 | 14 | 0.53 (0.27-1.00) | 0.051 | 13 | 1.67 (0.94-2.98) | 0.083 |
| ***High gases/fumes*** | 632 | 1.02 (0.91-1.13) | 0.767 | 409 | 1.02 (0.88-1.17) | 0.819 | 196 | 0.92 (0.77-1.13) | 0.458 | 102 | 0.97 (0.73-1.31) | 0.882 |
| *Men* | 438 | 1.14 (0.98-1.32) | 0.096 | 303 | 1.06 (0.9-1.26) | 0.48 | 149 | 1.06 (0.85-1.35) | 0.58 | 71 | 0.78 (0.53-1.15) | 0.205 |
| *Women* | 194 | 0.87 (0.74-1.03) | 0.11 | 106 | 0.96 (0.76-1.12_ | 0.74 | 47 | 0.77 (0.55-1.06) | 0.107 | 31 | 1.3 (0.83-2.04) | 0.249 |
| ***High herbicides*** | 49 | **1.74 (1.27-2.39)** | **0.001** | 34 | **1.47 (1.01-2.18)** | **0.048** | 14 | 1.48 (0.89-2.46) | 0.129 | 7 | 1.03 (0.53-2.01) | 0.915 |
| *Men* | 45 | **1.73 (1.24-2.41)** | **0.001** | 30 | **1.56 (1.01-2.42)** | **0.047** | 13 | 1.52 (0.86-2.68) | 0.148 | 6 | 1.2 (0.56-2.61) | 0.633 |
| *Women* | - | **-** | **-** | - | - | - | **-** | **-** | **-** | - | **-** | **-** |
| ***High insecticides*** | 76 | **1.53 (1.19-1.96)** | **0.001** | 53 | 1.36 (0.99-1.86) | 0.059 | 25 | 1.16 (0.72-1.89) | 0.533 | 11 | 0.91 (0.54-1.54) | 0.738 |
| *Men* | 68 | **1.6 (1.23-2.11)** | **0.001** | 47 | 1.35 (0.95-1.93) | 0.093 | 22 | 1.16 (0.71-1.89) | 0.559 | 10 | 0.85 (0.46-1.57) | 0.612 |
| *Women* | 8 | 1.24 (0.58-2.6) | 0.58 | 6 | 1.62 (0.81-3.28) | 0.173 | - | **-** | **-** | - | **-** | **-** |
| ***High fungicides*** | 81 | 1.42 (1.11-1.79) | 0.004 | 56 | 1.25 (0.94-1.68) | 0.129 | 25 | 1.08 (0.68-1.7) | 0.735 | 12 | 0.84 (0.51-1.39) | 0.51 |
| *Men* | 73 | 1.47 (1.14-1.89) | 0.003 | 50 | 1.22 (0.89-1.68) | 0.216 | 22 | 1.05 (0.67-1.66) | 0.827 | 11 | 0.82 (0.46-1.47) | 0.51 |
| *Women* | 8 | 1.23 (0.58-2.6) | 0.58 | 6 | 1.63 (0.81-3.28) | 0.173 | - | **-** | **-** | - | **-** | **-** |
| ***High aromatic solvents*** | 262 | **1.17 (1.01-1.38)** | **0.042** | 178 | 1.05 (0.87-1.27) | 0.617 | 82 | 1.08 (0.85-1.39) | 0.51 | 43 | 0.94 (0.62-1.41) | 0.761 |
| *Men* | 237 | 1.16 (0.97-1.37) | 0.099 | 159 | 1.01 (0.83-1.24) | 0.91 | 75 | 1.03 (0.78-1.35) | 0.81 | 36 | 0.79 (0.51-1.2) | 0.276 |
| *Women* | 25 | 1.25 (0.86-1.81) | 0.24 | 19 | 1.32 (0.86-2.03) | 0.202 | 7 | **1.91 (1.08-3.35)** | **0.024** | 7 | **2.31 (1.31-4.07)** | **0.004** |
| ***High chlorinated solvents*** | 191 | 1.13 (0.96-1.32) | 0.144 | 118 | 1.2 (0.99-1.45) | 0.061 | 55 | 1.17 (0.9-1.53) | 0.225 | 35 | 1.12 (0.76-1.64) | 0.561 |
| *Men* | 164 | 1.15 (0.97-1.39) | 0.113 | 99 | 1.19 (0.96-1.48) | 0.111 | 50 | 1.14 (0.85-1.54) | 0.383 | 27 | 0.87 (0.57-1.33) | 0.521 |
| *Women* | 27 | 0.93 (0.65-1.34) | 0.713 | 19 | 1.15 (0.78-1.72) | 0.481 | 5 | 1.55 (0.92-2.59) | 0.097 | 8 | **2.39 (1.38-4.16)** | **0.002** |
| ***High other solvents*** | 379 | 0.95 (0.85-1.08) | 0.492 | 241 | 0.97 (0.83-1.15) | 0.796 | 114 | 0.94 (0.75-1.17) | 0.563 | 58 | 0.92 (0.66-1.28) | 0.632 |
| *Men* | 243 | 1.06 (0.9-1.25) | 0.51 | 167 | 0.96 (0.79-1.17) | 0.669 | 78 | 0.98 (0.96-1.01) | 0.891 | 38 | 0.78 (0.52-1.18) | 0.234 |
| *Women* | 136 | 0.85 (0.7-1.04) | 0.12 | 74 | 1.04 (0.79-1.37) | 0.737 | 36 | 0.92 (0.63-1.33) | 0.653 | 20 | 1.14 (0.66-1.99) | 0.633 |

The model was adjusted for age, sex, smoking and education

This table excluded all dust, all pesticides, all solvents and all metals.

| **Occupational exposures** | **Disability free survival** | | | **All-cause mortality** | | | **Cancer-related mortality** | | | **CVD related mortality** | | | |
| --- | --- | --- | --- | --- | --- | --- | --- | --- | --- | --- | --- | --- | --- |
|  | **n*** | **HR (95% CI)** | **p-value** | **n** | **HR (95% CI)** | **p-value** | **n** | **HR (95% CI)** | **p-value** | **N** | **HR (95% CI)** | **p-value** | |
| ***High all dust exposures*** † | 151 | 1.17 (0.95-1.44) | 0.145 | 105 | 1.12 (0.87-1.44) | 0.365 | 48 | 1.19 (0.84-1.67) | 0.33 | 30 | 0.74 (0.42-1.29) | 0.297 | |
| Cumulative exposures in upper tertile‡ | 134 | 1.1 (0.87-1.39) | 0.42 | 95 | 1.03 (0.78-1.37) | 0.793 | 45 | 1.1 (0.75-1.6) | 0.632 | 27 | 0.68 (0.38-1.2) | 0.183 | |
|  | | | | | | | | | | | | | |
| ***High all pesticide exposure*** | 16 | 1.10 (0.66-1.87) | 0.71 | 11 | 1.21 (0.69-2.13) | 0.499 | 4 | 0.87 (0.46-1.67) | 0.691 | 2 | 0.49 (0.16-1.56) | 0.23 | |
| Cumulative exposures in upper tertile | 11 | 1.11 (0.59-2.07) | 0.738 | 9 | 1.32 (0.68-2.54) | 0.41 | 4 | 0.9 (0.48-1.72) | 0.77 | 1 | 0.42 (0.27-1.32) | 0.26 | |
|  | | | | | | | | | | | | |  |
| ***High all solvents exposure*** | 56 | 1.17 (0.88-1.54) | 0.275 | 30 | **1.58 (1.08-2.32)** | **0.019** | 11 | **2.28 (1.29-4.01)** | **0.004** | 11 | 1.46 (0.82-2.62) | 0.198 | |
| Cumulative exposures in upper tertile | 77 | 1.21 (0.99-1.48) | 0.059 | 49 | 1.08 (0.83-1.41) | 0.551 | 21 | 2.15 (0.78-1.71) | 0.48 | 16 | 1.29 (0.82-2.01) | 0.27 | |
| 0  .27 | | | | | | | | | | | | | |
| ***High all metals exposure*** | 42 | 1.35 (0.96-1.9) | 0.09 | 26 | **1.55 (1.01-2.39)** | **0.044** | 9 | **2.23 (1.24-4.04)** | **0.007** | 9 | 1.27 (0.68-2.38) | 0.45 | |
| Cumulative exposures in upper tertile | 37 | 1.22 (0.86-1.72) | 0.267 | 23 | 1.37 (0.88-2.13) | 0.16 | 8 | **2.47 (1.29-4.73)** | **0.004** | 8 | 1.16 (0.64-2.12) | 0.61 | |

**Table S9** Sensitivity analysis for the association between ever high and cumulative exposures and ASPREE endpoints in aspirin group (n=6070)

The model was adjusted for age, sex, smoking and education

**Table S10** Sensitivity analysis for the association between ever high and cumulative exposures and ASPREE endpoints in placebo group (n=6145)

| **Occupational exposures** | **Disability free survival** | | | **All-cause mortality** | | | **Cancer-related mortality** | | | **CVD related mortality** | | | |
| --- | --- | --- | --- | --- | --- | --- | --- | --- | --- | --- | --- | --- | --- |
|  | **n*** | **HR (95% CI)** | **p-value** | **n** | **HR (95% CI)** | **p-value** | **n** | **HR (95% CI)** | **p-value** | **N** | **HR (95% CI)** | **p-value** | |
| ***High all dust exposures*** † | 160 | **1.32 (1.08-1.59)** | **0.004** | 108 | 1.19 (0.94-1.49) | 0.148 | 52 | 1.07 (0.77-1.52) | 0.67 | 23 | 1.19 (0.76-1.870 | 0.45 | |
| Cumulative exposures in upper tertile‡ | 143 | **1.36 (1.1-1.67)** | **0.003** | 94 | **1.29 (1.01-1.66)** | **0.048** | 49 | 1.05 (0.73-1.51) | 0.79 | 19 | 1.47 (0.83-2.6) | 0.188 | |
|  | | | | | | | | | | | | | |
| ***High all pesticide exposure*** | 27 | **2.39 (1.69-3.39)** | **<**  **0.001** | 19 | 1.4 (0.83-2.38) | 0.21 | 9 | **1.94 (1.02-3.84)** | **0.049** | 3 | 1.07 (0.46-2.46) | 0.88 | |
| Cumulative exposures in upper tertile | 20 | **2.37 (1.5-3.75)** | **<**  **0.001** | 10 | **2.45 (1.3-4.61)** | **0.004** | 6 | 2.06 (0.77-5.54) | 0.151 | 2 | 1.62 (0.97-2.7) | 0.06 | |
|  | | | | | | | | | | | | |  |
| ***High all solvents exposure*** | 52 | 1.14 (0.9-1.44) | 0.269 | 30 | 1.32 (0.96-1.82) | 0.09 | 17 | 1.17 (0.79-1.7) | 0.43 | 7 | 1.41 (0.65-3.07) | 0.387 | |
| Cumulative exposures in upper tertile | 56 | 0.93 (0.75-1.15) | 0.519 | 36 | 0.97 (0.73-1.31) | 0.869 | 22 | 0.78 (0.53-1.17) | 0.238 | 7 | 1.48 (0.91-2.39) | 0.11 | |
|  | | | | | | | | | | | | | |
| ***High all metals exposure*** | 41 | 1.24 (0.93-1.67) | 0.144 | 24 | 1.27 (0.86-1.88) | 0.23 | 12 | 1.03 (0.64-1.66) | 0.89 | 8 | 1.74 (0.82-3.7) | 0.146 | |
| Cumulative exposures in upper tertile | 36 | 1.08 (0.81-1.44) | 0.58 | 21 | 1.04 (0.72-1.52) | 0.81 | 10 | 0.92 (0.56-1.52) | 0.75 | 7 | 1.23 (0.59-2.55) | 0.58 | |

The model was adjusted for age, sex, smoking and education

**Table S11** Association for the low and high exposure categories compared to same non-exposure reference category and ASPREE

| **Occupational exposures** | **Disability free survival** | | | **All-cause mortality** | | | **Cancer-related mortality** | | | **CVD related mortality** | | |
| --- | --- | --- | --- | --- | --- | --- | --- | --- | --- | --- | --- | --- |
|  | **n=**  **6099** | **HR (95% CI)** | **p-value** | **n=**  **6447** | **HR (95% CI)** | **p-value** | **n** | **HR (95% CI)** | **p-value** | **n** | **HR (95% CI)** | **p-value** |
| ***No exposure*** | 883 | Ref. | - | 535 | Ref. | - | 278 | Ref. | - | 121 | Ref. | - |
| ***Low all dust exposures*** | 435 | 0.9 (0.8-1.00) | 0.07 | 259 | 1.01 (0.88-1.16) | 0.93 | 125 | 0.86 (0.69-1.06) | 0.146 | 66 | 0.99 (0.73-1.34) | 0.953 |
| ***High all dust*** | 311 | **1.14 (1.07-1.39)** | **0.004** | 213 | 1.10 (0.92-1.32) | 0.273 | 100 | 1.06 (0.82-1.36) | 0.648 | 53 | 1.01 (0.71-1.42) | 0.99 |
|  | | | | | | | | | | | | |
| ***No exposure*** | 883 | Ref. | - | 535 | Ref. | - | 278 | Ref. | - | 121 | Ref. | - |
| ***Low all pesticides*** | 43 | 1.24 (0.92-1.67) | 0.155 | 30 | 1.3 (0.88-1.93) | 0.188 | 14 | 1.01 (0.5-2.03) | 0.979 | 8 | 0.89 (0.48-1.65) | 0.72 |
| ***High all pesticides*** | 43 | **1.71 (1.24-2.37)** | **0.001** | 30 | 1.28 (0.78-1.78) | 0.424 | 13 | 1.36 (0.83-2.23) | 0.216 | 5 | 0.84 (0.44-1.57) | 0.584 |
|  | | | | | | | | | | | | |
| ***No exposure*** | 883 | Ref. | - | 535 | Ref. | - | 278 | Ref. | - | 121 | Ref. | - |
| ***Low all solvents*** | 322 | 0.95 (0.83-1.08) | 0.44 | 215 | 0.96 (0.82-1.12) | 0.594 | 102 | 0.91 (0.72-1.16) | 0.477 | 46 | 0.78 (0.55-1.12) | 0.178 |
| ***High all solvents*** | 108 | 1.14 (0.94-1.37) | 0.188 | 60 | **1.39 (1.08-1.78)** | **0.008** | 28 | 1.28 (0.90-1.81) | 0.164 | 18 | 1.45 (0.89-2.39) | 0.138 |
|  | | | | | | | | | | | | |
| ***No exposure*** | 883 | Ref. | - | 535 | Ref. | - | 278 | Ref. | - | 121 | Ref. | - |
| ***Low all metals*** | 105 | 1.02 (0.84-1.24) | 0.837 | 76 | 0.91 (0.73-1.12) | 0.355 | 33 | 1.01 (0.74-1.39) | 0.94 | 20 | 0.52 (0.32-0.86) | 0.01 |
| ***High all metals*** | 83 | **1.29 (1.03-1.63)** | **0.023** | 50 | 1.35 (0.94-1.64) | 0.115 | 21 | 1.26 (0.82-1.92) | 0.287 | 17 | 1.34 (0.79-2.25) | 0.269 |

The model was adjusted for age, sex, smoking and education
